# Research on Alkali-Activated, Spinelized Kaolin Cementitious Composite Materials

**DOI:** 10.3390/ma18174147

**Published:** 2025-09-04

**Authors:** Yuyang Feng, Chenyi Gao, Feng Yuan, Jun Sun, Qijiang Li

**Affiliations:** 1 Research Center of Ancient Ceramic, Jingdezhen Ceramic University, Jingdezhen 333001, China; 2420091003@stu.jcu.edu.cn (Y.F.); 2410024005@stu.jcu.edu.cn (C.G.); yuanfeng@jcu.edu.cn (F.Y.); 2010024004@stu.jcu.edu.cn (J.S.); 2 Jiangxi Ceramic Heritage Conservation and Imperial Kiln Research Collaborative Innovation Center, Jingdezhen 333001, China

**Keywords:** alkali activation, curing temperature, curing time, zeolite-like

## Abstract

This study prepared alkali-activated cementitious composites using high-whiteness kaolin, sodium water glass, and NaOH as the main raw materials. Multiple methods, including FE-SEM, XRD, whiteness/light transmittance tests, shrinkage rate measurements, DSC-TG, flexural strength testing, and hydrolysis resistance testing, were used to investigate the effects of curing temperature and time on material properties. The optimal parameters were determined as kaolin calcined at 1100 °C, activator modulus 1.25, calcined kaolin-to-activator ratio 1:1, and 2.5% deionized water added for molding. The optimal sample achieved a flexural strength of 23.81 MPa, with the bonding strength to porcelain 60.17 times that of gypsum and 1.90 times that of kaolin-bonded materials. Curing below 100 °C slowed polymerization, while temperatures exceeding 100 °C accelerated it, with violent reaction at 120 °C. Curing beyond 10 h reduced flexural strength. A large number of cage-like, ‘zeolite-like’ structures formed, closely relating to material properties. This study provides references for ceramic restoration materials.

## 1. Introduction

The reserves of ceramic cultural relics are substantial, yet their statuses of protection, restoration, exhibition, and utilization are not optimistic. The restoration of ceramic cultural relics can not only improve their aesthetic appearance but also enhance people’s in-depth understanding of cultural relics. In the current literature, the most prevalent ceramic patching materials are primarily gypsum and epoxy resin. However, gypsum exhibits a loose structure, inadequate strength, and a high water absorption rate, whilst also lacking the texture of porcelain. Epoxy resin has problems such as aging, discoloration, and poor durability, making it difficult to meet the long-term protection requirements of ancient ceramics [1,2,3,4]. As a body-filling material for ancient ceramic restoration, it is required to be curable at low temperatures (≤200 °C) while possessing high strength and aging resistance. Alkali-activated cementitious material is expected to be a promising ancient ceramic restoration material due to its good bonding performance, chemical stability, and a mineral composition that is similar to that of ceramic materials [5,6,7].

Alkali-activated cementitious materials have a wide range of applications. Some studies have already attempted to apply alkali-activated cementitious materials to the restoration practice of ancient ceramics. Li Qijiang proposed applying alkali-activated, metakaolin-based cementitious composite materials to the restoration of ancient ceramics, and their performance was superior to that of gypsum-based body-filling materials [8]. In the research on reaction conditions, curing temperature and curing time are key factors. Bian Libo et al. conducted a study which demonstrated that the curing system directly determines the structure and properties of alkali-activated cementitious materials [9]. Ye Jiaolong proposed that an appropriate curing system can enhance the degree of polymerization reaction of alkali-activated cementitious materials, effectively improving their mechanical properties and dry-shrinkage characteristics [10]. In addition, the functional development and intelligent prediction of alkali-activated cementitious materials have also become new research priorities [11].

Although some progress has been made in the research on alkali-activated cementitious materials in various fields [12,13,14,15,16,17,18,19,20,21,22], their application in the field of ancient ceramic restoration is still in the exploration stage. In this study, silicon–aluminum spinelized kaolin was used as the raw material to prepare high-performance, alkali-activated cementitious composite materials, and we deeply explored the influence of factors such as the curing temperature and curing time on the material properties [23], aiming to provide a more solid theoretical and practical basis for the wide application of alkali-activated cementitious materials in the field of ancient ceramic restoration.

## 2. Experimental Content

### 2.1. Experimental Raw Materials

This experiment used high-whiteness kaolin as the aluminosilicate raw material, and its chemical composition is shown in Table 1. Sodium water glass produced by Shandong Yousuo Chemical Technology Co., Ltd. (Linyi, China) was used as the activator. It had a Baumé degree of 40′Be, a modulus of 3.3, and a solid-phase content of 34%. Flake-shaped NaOH produced by Tianjin Hengxing Chemical Reagent Manufacturing Co., Ltd. (Tianjin, China) was used to adjust the modulus and pH of the water glass. It was of analytical grade, containing more than 94% sodium hydroxide.

### 2.2. Testing Methods

The microstructure of the samples was tested using an SU-8010 field emission scanning electron microscope produced by HITACHI, Hitachi, Japan. The measurement voltage was 20 KV, and the spot diameter was 5.0. The TG-DSC of kaolin was tested using an STA449C comprehensive thermal analyzer produced by Netzsch, Selb, Germany. The sample was tested from room temperature to 1300 °C at a heating rate of 15 °C/min. The powder samples were weighed at 10–15 mg each time, and the test atmosphere was argon. The infrared absorption spectrum data of the samples were tested using a Nicolet 5700 Fourier-transform infrared spectrometer produced by Thermo Fisher Scientific, Waltham, MA, USA. The resolution was 1 cm^−1^, and the wavenumber range was 400–4000 cm^−1^.

The phase composition of the prepared samples was analyzed using the D8-Advance X-ray diffractometer from Bruker, Berlin, Germany. The CuKα radiation parameters were set at 100 mA tube current and 40 kV tube voltage, with a scanning angle range of 5 ≤ 2θ ≤ 70. The whiteness and transmittance of the prepared samples were measured using a fluorescence whiteness meter and a photoelectric haze meter.

The flexural strength was tested using a microcomputer-controlled universal testing machine (Jinan Yongke Experimental Instrument Co., Ltd., located in Jinan, China), with a constant loading speed of 0.5 mm/min on average. The test samples measured 50 mm × 20 mm × 13 mm. Dimensions varied slightly between samples due to the shrinkage rate. Seven samples were tested, with the average value calculated. The test method for the hydrolysis resistance of the samples was as follows: The experimental samples were dried in an oven at 105 °C for 2 h, then taken out and weighed to record the dried mass m_0_ of each experimental sample. Subsequently, each experimental sample was placed in a numbered covered container, and deionized water was added to the container at 20 times the dried mass of each sample. The container mouth was covered to prevent water evaporation. Then, the pH values of the samples after soaking for 1 day and 14 days were tested [24,25]. The pH of the aqueous solution was measured using a pH meter (Shanghai Yidian Technology Instrument Co., Ltd., located in Shanghai, China).

### 2.3. Kaolin Pretreatment

**Figure 1:**
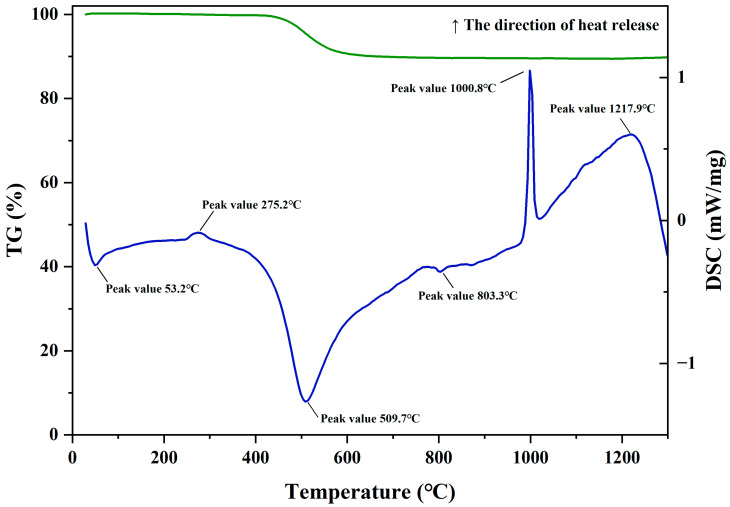
TG-DSC curve of Dehua kaolin.

The TG-DSC test curve of kaolin is shown in Figure 1. It can be seen from the figure that the DSC curve displays 4 obvious peaks. The peaks near 53.2 °C and 509.7 °C are endothermic peaks, and the peaks near 1000.8 °C and 1217.9 °C are exothermic peaks. There are also two weak peaks, an exothermic peak near 275.2 °C and an endothermic peak at 803.3 °C. The endothermic peak near 53.2 °C is due to the removal of a small amount of free water. The exothermic peak near 275.2 °C is caused by the combustion of a small amount of organic matter in the kaolin [26,27]. The intense endothermic reaction near 509.7 °C is mainly caused by the removal of structural water from the mineral phase of kaolinite to transform into amorphous metakaolin, and there is also an endothermic contribution from the transformation of α-quartz to β-quartz at 573 °C. However, well-crystallized kaolinite still retains some residual structure and a small amount of structural water after passing through the intense endothermic peak, showing a gradually rising trend on the curve and forming a very weak endothermic peak near 803.3 °C. This process continues until the temperature reaches 950 °C. The intense and sharp exothermic peak near 1000.8 °C is formed by the heat release during the transformation of amorphous metakaolin into silicon–aluminum spinel and mullite-like phases. This is a rapid and intense exothermic effect, which is a characteristic of the differential thermal curve of well-crystallized kaolinite [26]. During this process, amorphous SiO_2_ is also generated [27]. The broad and gentle exothermic peak near 1217.9 °C is due to the continuous formation of mullite [28]. The TG curve shows that the calcination weight loss of kaolin is mainly caused by the removal of structural water from kaolinite, and the weight-loss temperature is concentrated in the range of 450–650 °C. Due to the removal of structural water, the structure of kaolinite is broken, and it is transformed into active metakaolin. Its activity is mainly generated by structural reorganization and broken bonds, and the reaction itself does not produce independent Al_2_O_3_ or SiO_2_ phases. During the transformation process of metakaolin to a stable phase under high-temperature action, SiO_2_ segregation occurs in each reaction step, forming a gradual and continuous process. Therefore, during the calcination process of kaolin, kaolin not only generates pozzolanic activity but also generates amorphous active SiO_2_.

It is generally believed that kaolin transforms into amorphous metakaolin at 600 °C. As the temperature continues to rise, the alkali-activation activity of metakaolin gradually increases. When the calcination temperature is 600–800 °C, metakaolin shows strong alkali-activation activity and possesses a loose structure. When the calcination temperature exceeds 800 °C, amorphous metakaolin transforms into a lattice-stable mullite phase, the structure re-aggregates, and its activity shows a downward trend, hindering the reaction process of alkali-activated cementitious materials. However, the Si-Al spinel in kaolin is a transitional form to mullite, which is relatively unstable. The silicon–oxygen structure and aluminum–oxygen structure of Si-Al spinel change greatly compared with the structure of metakaolin, causing some Al_2_O_3_ to lose activity and forming SiO_2_ segregation. Under certain conditions, it can still generate activation activity [29,30].

### 2.4. Experimental Process

According to the TG-DSC curve, silicon–aluminum spinel forms at 1000 °C. However, actual calcination shows a phase transformation lag due to the raw material batch size and heating rate effects. Moreover, alkali-activated cementitious materials prepared from 1200 °C-calcined raw materials exhibited expansion during curing. In this experiment, kaolin calcined at 1100 °C was selected as the aluminosilicate raw material. Sodium hydroxide and sodium water glass were used to prepare the activator with a modulus of 1.25. The ratio of calcined kaolin to the activator was 1:1, and 2.5% deionized water was added to adjust its molding performance. Alkali-activated cementitious composite materials were prepared under a certain curing system.

## 3. Results and Discussion

### 3.1. Influence of Curing Temperature on Sample Properties

The curing temperatures selected in this single-factor experiment were 60 °C, 80 °C, 100 °C, 110 °C, 120 °C, and 130 °C. The initial curing time was 10 h. The abbreviation JYW stands for the pinyin initials of “spinel-based cementitious material curing temperature” in Chinese, with the numbers following the abbreviation representing the specimen’s curing temperature.

**Figure 2:**
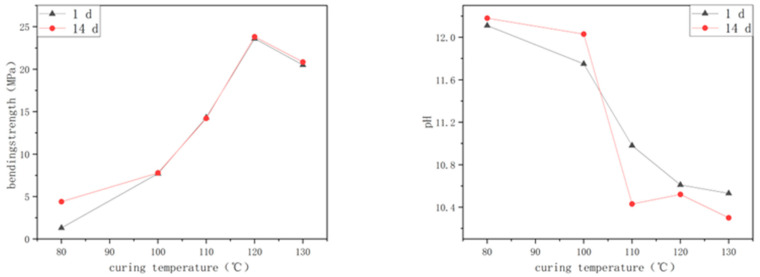
Effect of curing temperature on flexural strength and hydrolysis resistance of samples.

The curing temperature has an important influence on the properties of alkali-activated, spinelized, kaolin-based cementitious composite materials. When cured at 60 °C, the alkali does not easily activate spinelized kaolin, and the sample undergoes a slow polymerization reaction and cannot be cured after 24 h of curing. Figure 2 shows a line chart of the influence of the curing temperature on the flexural strength and hydrolysis resistance of the cementitious composite materials. It can be seen from Figure 2 that above 80 °C, as the curing temperature gradually increases, the 1-day flexural strength of the prepared samples gradually increases. After curing at 100 °C, the growth rate becomes faster. When cured at 120 °C, the flexural strength can reach 23.60 MPa, which is 3.06 times that of the samples prepared at 100 °C. Comparing with samples that were cured at 120 °C, those that were cured at 130 °C exhibited a marginally reduced flexural strength. The experimental results show that the polymerization reaction of alkali-activated, spinelized kaolin cementitious composite materials is rapid and complete at 120 °C and above. Compared with the 1-day flexural strength, for the samples prepared at 80 °C, the 14-day flexural strength increased by 2.36 times. For the samples prepared above 100 °C, the 14-day flexural strength did not change. The slight fluctuations in the flexural strength data are related to the pores and micro-cracks inside different samples. Therefore, the initial temperature at which the alkali-activated, spinelized kaolin cementitious composite materials can react is around 80 °C. Increasing the curing temperature will accelerate the rate of the polymerization reaction. Above 120 °C, the polymerization reaction proceeds violently, forming a dense, high-degree-of-polymerization network-like aluminosilicate substance, and meaning the final shrinkage rate of the JYW120 sample reaches 12.04%, which is similar to the total shrinkage rate of general white-bodied porcelain after firing. Therefore, the flexural strength of the prepared samples can be greatly improved.

Figure 2 shows the change law of the hydrolysis solution sample’s pH under different curing temperatures, which demonstrates that as the curing temperature gradually increases, the change in the pH of the 1-day hydrolysis solution samples gradually decreases. The sample cured at 120 °C has a 1.14 lower pH in the hydrolysis solution than the sample cured at 100 °C, and the concentration of alkali in the hydrolysis solution is reduced by more than 10 times. This is opposite to the change law of the flexural strength of the samples with the curing temperature, but the internal reasons are the same. The higher the curing temperature, the more complete the polymerization reaction. And accordingly, the more high-polymer network-like structures are generated inside the sample, making it easier for the unreacted OH^−^ to be sealed in the network structure, thus reducing the change in the pH of the hydrolysis solution. The change laws of the 14-day pH and the 1-day pH are different. For the samples cured at 80 °C and 100 °C, due to the incomplete polymerization reaction, the amount of residual activator that has not participated in the reaction is high, meaning the pH of the sample aqueous solution still increases after 14 days. For the samples cured above 110 °C, the pH of the aqueous solution after 14 days decreases, which is related to the re-adsorption of OH^−^ by the sample in the aqueous solution. In the long-term pH detection and observation of the aqueous solution of this group of samples, the pH shows a regular alternating change law of decreasing, increasing, and decreasing. This law shows that the change in the pH of the sample aqueous solution is not only affected by the structure of the sample itself but also (and mainly) related to the residual OH^−^ on the surface of the sample. That is, the OH^−^ migrating from the sample to the aqueous solution is mainly from the surface of the sample, and very few OH^−^ migrate from the inside and the surface to the aqueous solution during subsequent soaking, reflecting the stability of the sample structure.

In this experiment, the JYW130 sample had a certain degree of deformation, resulting in a larger shrinkage rate than the JYW120 sample. The reason is that the polymerization reaction of the sample at 130 °C was too intense, and the volatilization rates of water molecules on the surface and the bottom of the sample in the mold were different, resulting in differences in the polymerization reaction rates on the surface and the bottom, causing inconsistent shrinkage and a certain degree of deformation. Therefore, the appropriate curing temperature for preparing alkali-activated, spinelized kaolin cementitious composite materials is 120 °C.

### 3.2. Influence of Curing Time on Sample Properties

The duration of the curing time affects the degree of the polymerization reaction of the prepared material, especially the change in its later-stage properties. The curing times selected in this single-factor experiment were 6 h, 10 h, 16 h, 24 h, and 34 h.

**Figure 3:**
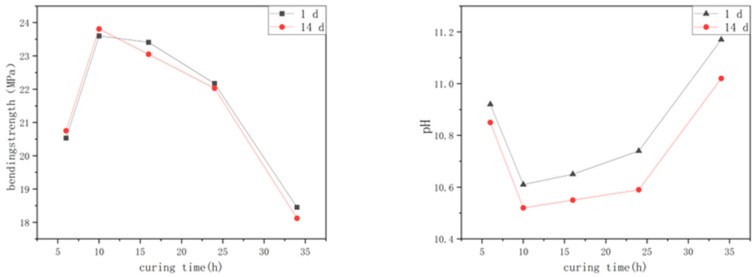
Effect of curing time on flexural strength and hydrolysis resistance of samples.

The experimental results of preparing alkali-activated, spinelized kaolin cementitious composite materials by curing at 120 °C for different times are shown in Figure 3.

The line chart about the influence of different curing times on the samples’ flexural in Figure 3 displays that as the curing time gradually increases, the 1-day flexural strength of the cementitious composite material samples exhibits an initial increase and subsequent decrease as the curing time gradually increases. At this point, the flexural strength of the optimal sample can reach 23.81 MPa. The increase in the flexural strength is clear in the early stage of curing, but when the curing time exceeds 10 h, the flexural strength of the sample begins to decrease. The change law of the 14-day flexural strength is the same as that of the 1-day flexural strength, but for the samples cured for more than 16 h, the 14-day flexural strength value is slightly lower than its 1-day flexural strength. This indicates that the polymerization reaction of the cementitious composite material prepared by curing alkali-activated, spinelized kaolin at 120 °C for 10 h has reached the optimal degree. Continuous curing at high temperatures causes the internal polymerization reaction of the sample to continue, and the polymer cage-like particles are gradually damaged, reducing the flexural strength.

Ceramic bonding materials require an excellent adhesion strength to ceramics. Insufficient bonding strength risks detachment of repaired areas, compromising restoration outcomes. This study employed flexural strength as the evaluation metric for bonding performance in alkali-activated spinel, petrochemical cementitious polymer materials.

Porcelain specimens were prepared from Jingdezhen white clay and fired at 1300 °C, and we applied external force to create a natural fracture surface. Next, specimens were bonded using JYW120 adhesive material, cured to form bonded specimens, and tested, yielding a flexural strength of 24.67 MPa, and demonstrating an exceptional bonding performance suitable for ceramic adhesion applications. The optimal bonded ceramic body demonstrated exceptional strength, with its bonding performance reaching 60.17 times that of gypsum and 1.90 times that of kaolin-based binders. Comprehensive evaluations confirmed the JYW120 adhesive polymer material’s superior bonding strength over both gypsum and kaolin-based alternatives, establishing it as the superior choice among the three materials.

Based on the experimental results, we prepared sample JYY2 gel-coated polymer using the optimal formula and tested its whiteness, light transmittance, and shrinkage rate. The abbreviation JYY stands for the pinyin initials of the optimal curing system application for spinel-based cementitious materials in Chinese. The test results showed that JYY2 achieved a light transmittance of 31.8% and a whiteness of 61.6%, comparable to porcelain properties. However, the significant shrinkage rate may hinder its application in ceramic restoration. Nevertheless, our practical experiments demonstrated that multiple restoration procedures can be performed to achieve effective ceramic restoration. Furthermore, future research should focus on improving shrinkage performance to enhance its practical application in ceramic restoration.

### 3.3. Analysis of the Formation Mechanism of Polymeric Materials

**Figure 4:**
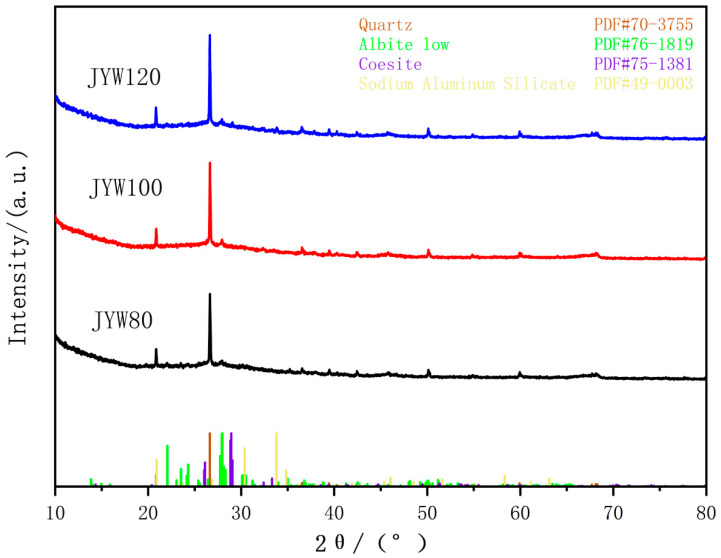
XRD patterns of alkali-activated gelling polymer with spinel kaolin at different curing temperatures.

The XRD patterns of JYW80, JYW100, and JYW120, a high-kaolin, gel-cemented material activated by alkali, under different curing temperatures are shown in Figure 4.

As demonstrated in Figure 4, the diffraction patterns of JYW80 and JYW100 are essentially identical, with no new phases formed. However, JYW120 exhibits distinct feldspar and sodium aluminosilicate diffraction peaks. In the diffraction pattern of JYW120, the feldspar (PDF#75-1381) shows three prominent strong lines at 2θ positions of 28.932°, 28.851°, and 29.060° on the (200), (100), and (002) planes, respectively. These lines cluster together as indicated by the standard mark in the figure. The sodium aluminosilicate (PDF#49-0003, Na_1.95_Al_1.95_Si_0.05_O_4_) shows a prominent strongest line at 2θ position 33.803° on the (200) plane. This indicates that under 120 °C alkaline excitation, the activator reacts with active SiO_2_ and Al_2_O_3_ to form the sodium aluminosilicate crystal [31]. Furthermore, the alkaline excitation promotes the transformation of amorphous SiO_2_ into feldspar. In the XRD patterns of JYW80, JYW100, and JYW120, only the strongest diffraction peak (040) plane of the albite crystal remains present.

**Figure 5:**
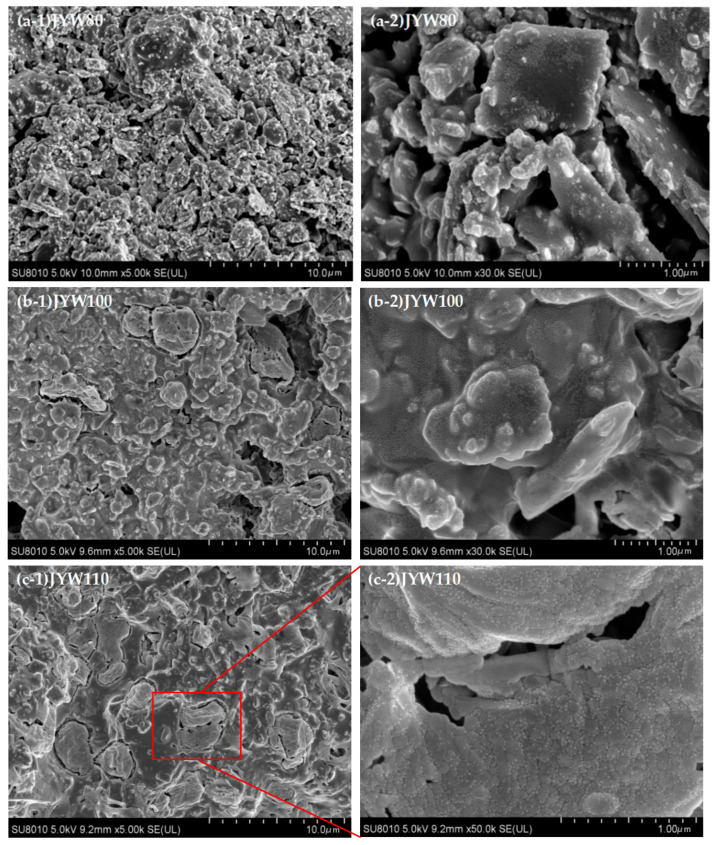

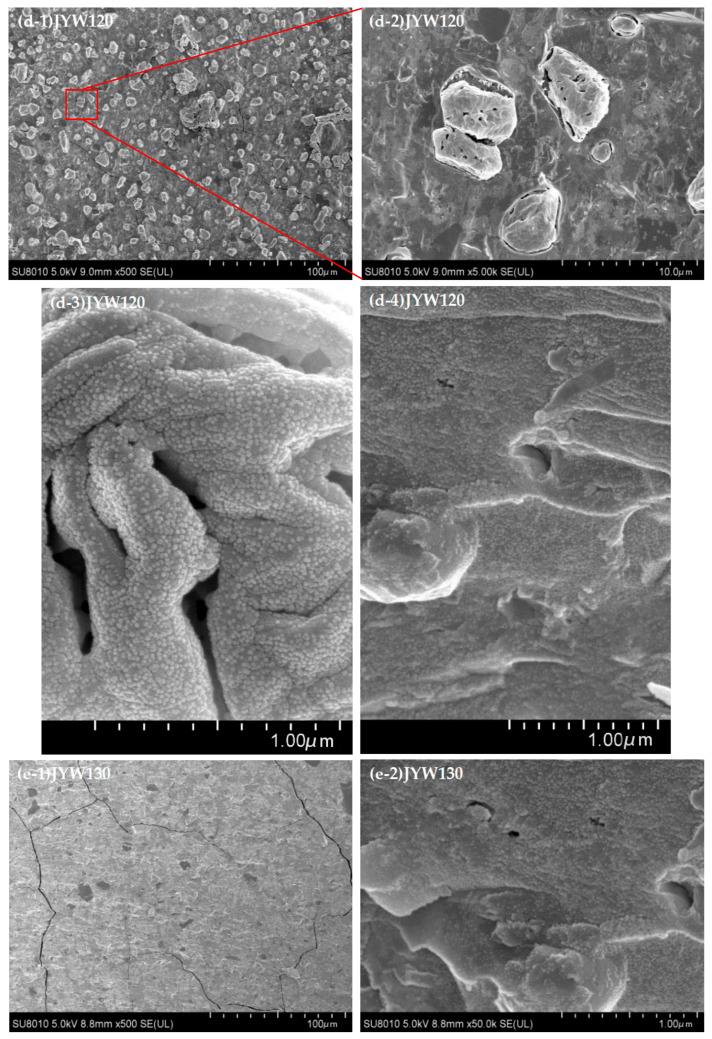
SEM images of alkali-activated materials with spinel kaolin at different curing temperatures.

It can be seen from Figure 5 that the gelling polymerization products at 80 °C, 100 °C, 110 °C, 120 °C, and 130 °C show distinct differences, and the spinelized kaolin morphology particles became invisible in the gelling polymeric material samples prepared by curing at 120 °C and above. The SEM morphology diagram of the sample section in Figure 5(a-1,a-2) JYW80 clearly shows the morphology of the spinelized kaolin particles, with the alkali activator uniformly covering its surface. The accumulation between the kaolin particles is not tight, and the porosity is large. In the SEM topography diagram of the JYW100 sample section (Figure 5(b-1,b-2)), the morphology of spinelized kaolin is still clearly visible. The alkali activator uniformly covers the surface of the spinelized kaolin particles and binds them tightly together. A layer of dense, small, granular, gelatinized polymeric substances of about 10 nm is formed on the surface of the particles, and cage-shaped, ‘zeolite-like’ structures with a size of about 5 μm are formed in some areas [32,33]. The morphology of spinelized kaolin is difficult to identify in the SEM topography diagram of the sample section in Figure 5(c-1,c-2) for JYW110. The main substance in the sample is gelatinized polymer, and the cage-shaped ‘zeolite-like’ structures have increased significantly. It is about 3 μm in size and there are micro-cracks in the material. The ‘zeolite-like’ substances consist of caged polymer particles measuring approximately 100 nm, with a row of smaller particles measuring around 10 nm distributed at the particle interface, as illustrated in Figure 5(c-2). In the SEM topography diagram of the sample section in Figure 5(d-4) for JYW120, a large number of caged ‘zeolite-like stones’ of about 5 μm in size are embedded in the seamless cementitious polymeric material. The dark gray area in the figure is quartz of different shapes, and micro-cracks can also be seen in the figure compared with the JYW110 sample, while the surface of the cage-shaped, ‘zeolite like’ structure is densely covered with a layer of small-particle, cementitious polymer material of around 10 nm, not just at the interface of particle accumulation, such as in Figure 5(d-3). The area without a cage-like structure is also covered with a layer of small particles of gelatinous polymer material measuring around 10 nm on its surface, such as in Figure 5(d-4). In Figure 5(e-1,e-2), the SEM morphology of the JYW130 sample section shows that only a small amount of quartz is uniformly distributed in the cementitious polymer material, and there is no cage-shaped, ‘zeolite-like’ structure. The material is dense but has wide micro-cracks. The surface of the material is covered with a layer of small-particle, cementitious polymer material with a diameter of about 10 nm.

**Figure 6:**
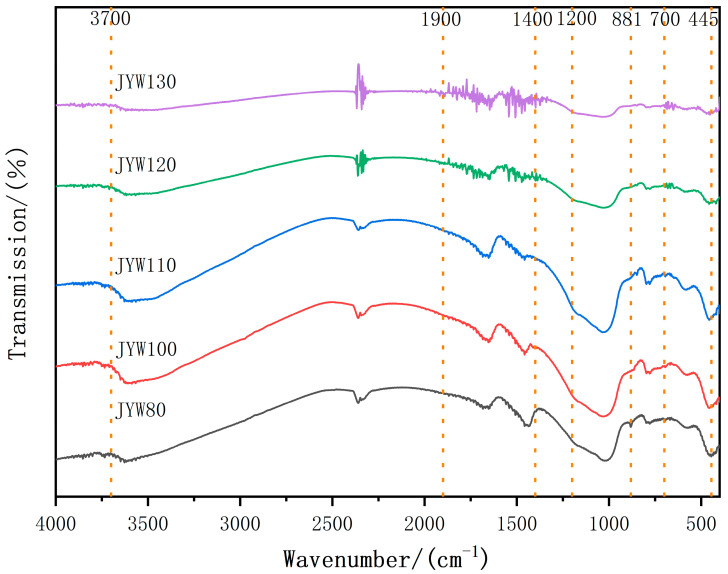
FTIR curves of alkali-activated gelling polymer with spinel kaolin at different curing temperatures.

Figure 6 shows the FTIR spectra of alkali-activated, spinelized kaolin cementitious composite materials under different curing temperatures. When cured at 80 °C, under the action of the activator, the Si-O and Si-O-Si stretching vibration absorption bands of kaolin in the range of 1200 cm^−1^–1000 cm^−1^ shift to lower wavenumbers, and an absorption band at 881 cm^−1^ is formed. A depolymerization reaction mainly occurs, generating a large amount of low-polymerized [SiO_4_] and isolated [SiO_4_]. The sharpening of the Si-O and Si-O-Al bending vibration absorption bands in the absorption band of 800 cm^−1^–400 cm^−1^ also reflects that a certain degree of polycondensation occurs in the [SiO_4_] and [AlO_6_] ion groups, but with a low degree of polymerization as the main feature. This is consistent with the results presented by SEM. The absorption band of 1900 cm^−1^–1400 cm^−1^ is the bending vibration caused by the adsorbed water present during the reaction process, and the diffuse and small absorption band of 4000 cm^−1^–3700 cm^−1^ is caused by the stretching vibration of free water. With the increase in the curing temperature, the absorption band at 881 cm^−1^ gradually disappears, the Si-O and Si-O-Si stretching vibration absorption bands in the range of 1200–1000 cm^−1^ shift to higher wavenumbers, and the Si-O bending vibration absorption band near 445 cm^−1^ shifts to a higher frequency and broadens, gradually forming [SiO_4_] ion groups with a high degree of polymerization. When cured at above 120 °C, the ‘zeolite-like’ Si-O bending vibration absorption band characteristics appear in the absorption band of 600–400 cm^−1^. A series of sharp absorption bands are generated in the absorption bands of 1900–1400 cm^−1^ and 700–630 cm^−1^, reflecting that a large amount of adsorbed water participates in the ‘zeolite-like’ polymerization reaction and is affected by the forces of different groups.

## 4. Conclusions

(1) After the high-temperature calcination of kaolin transforms into silicon–aluminum spinel, a substance with high Si/Al activity is formed, and a high activation temperature is required. The technological parameters for preparing the alkali-activated, spinelized kaolin gel polymer material are as follows: the kaolin is calcined at 1100 °C; the activator’s modulus is 1.25; the ratio of calcined kaolin to the activator is 1:1; 2.5% deionized water is added to adjust its molding performance; and it is cured at 120 °C for 10 h. The flexural strength of the material prepared according to these parameters can reach 23.81 MPa.
(2) SEM result shows that the curing temperature significantly affects the alkali-activated, spinelized kaolin gel polymer material. The alkali-activated reaction is incomplete below 120 °C. A cage-like, zeolite-like structure forms at 120 °C but disappears again when the temperature exceeds 120 °C. The FTIR results indicate that water participates in a deeper reaction, generating a water-containing, zeolite-like phase. Therefore, the gel polymer is mainly composed of high-polymerization-degree [SiO_4_]. The higher the curing temperature, the greater the quantity of high-polymerization-degree gel polymers generated.
(3) The alkaline-excited, spinel gel coagulation material prepared in this experiment not only provides a new high-performance inorganic gelling material with broad application prospects for the field of ceramic body filling but also provides some inspiration for the research on building materials.

## Figures and Tables

**Table 1:** Chemical composition of the kaolin clays.

Kind	SiO_2_	Al_2_O_3_	Fe_2_O_3_	TiO_2_	CaO	MgO	K_2_O	Na_2_O
Kaolin	56.43	40.69	0.16	0.06	0.15	0.47	0.52	1.51

## Data Availability

The original contributions presented in this study are included in the article. Further inquiries can be directed to the corresponding author.
